# Initial Phase of Anthracycline Cardiotoxicity Involves Cardiac Fibroblasts Activation and Metabolic Switch

**DOI:** 10.3390/cancers16010053

**Published:** 2023-12-21

**Authors:** Marialucia Telesca, Maria Donniacuo, Gabriella Bellocchio, Maria Antonietta Riemma, Elena Mele, Carmela Dell’Aversana, Giulia Sgueglia, Eleonora Cianflone, Donato Cappetta, Daniele Torella, Lucia Altucci, Giuseppe Castaldo, Francesco Rossi, Liberato Berrino, Konrad Urbanek, Antonella De Angelis

**Affiliations:** 1 Department of Experimental Medicine, University of Campania “Luigi Vanvitelli”, Via Costantinopoli 16, 80138 Naples, Italy; marialucia.telesca@unicampania.it (M.T.); maria.donniacuo@unicampania.it (M.D.); gabriella.bellocchio@unicampania.it (G.B.); mariaantonietta.riemma@unicampania.it (M.A.R.); elena.mele@unicampania.it (E.M.); francesco.rossi@unicampania.it (F.R.); liberato.berrino@unicampania.it (L.B.); antonella.deangelis@unicampania.it (A.D.A.); 2 Department of Precision Medicine, University of Campania “Luigi Vanvitelli”, Via Costantinopoli 16, 80138 Naples, Italy; carmela.dellaversana@unicampania.it (C.D.); giulia.sgueglia@unicampania.it (G.S.); lucia.altucci@unicampania.it (L.A.); 3 BIOGEM, Via Camporeale, 83031 Ariano Irpino, Italy; 4 Department of Medical and Surgical Sciences, Magna Graecia University, Viale Europa, 88100 Catanzaro, Italy; cianflone@unicz.it; 5 Department of Biological and Environmental Sciences and Technologies, University of Salento, Via Lecce-Monteroni, 73047 Lecce, Italy; donato.cappetta@unisalento.it; 6 Department of Experimental and Clinical Medicine, Magna Graecia University, 88100 Catanzaro, Italy; dtorella@unicz.it; 7 Institute of Experimental Endocrinology and Oncology “Gaetano Salvatore” (IEOS)-National Research Council (CNR), 80131 Naples, Italy; 8 Department of Molecular Medicine and Medical Biotechnologies, University of Naples “Federico II”, Via A. Pansini 5, 80131 Naples, Italy; giuseppe.castaldo@unina.it; 9 CEINGE-Advanced Biotechnologies “Franco Salvatore”, Via G. Salvatore 486, 80131 Naples, Italy

**Keywords:** text doxorubicin, cardiotoxicity, cardiac fibroblasts, phenotype switch

## Abstract

**Simple Summary:**

One of the oldest anticancer drugs in use is doxorubicin. It associates with cardiotoxicity that until now remains unresolved. Diastolic cardiac dysfunction can be the first sign, and develops at the very low doses. We asked whether short-term changes were preceded by even earlier effects. We tested functional and molecular changes in cardiac fibroblasts after very brief exposure to doxorubicin. Cardiac fibroblasts increased energy metabolism and differentiated into myofibroblast, expressing markers of cell activation and phenotype change. These results demonstrate that cardiac fibroblasts are effector cells that swiftly react to systemically administered drug. From the very first contact with doxorubicin, they can actively contribute to the initial phase of pathological myocardial remodeling. Thus, it is conceivable to foresee these cells as a target to counteract the cardiotoxicity of doxorubicin at the very early stage.

**Abstract:**

The application of doxorubicin (DOX) is hampered by cardiotoxicity, with diastolic dysfunction as the earliest manifestation. Fibrosis leads to impaired relaxation, but the mechanisms that operate shortly after DOX exposure are not clear. We asked whether the activation of cardiac fibroblasts (CFs) anticipates myocardial dysfunction and evaluated the effects of DOX on CF metabolism. CFs were isolated from the hearts of rats after the first injection of DOX. In another experiment, CFs were exposed to DOX in vitro. Cell phenotype and metabolism were determined. Early effects of DOX consisted of diastolic dysfunction and unchanged ejection fraction. Markers of pro-fibrotic remodeling and evidence of CF transformation were present immediately after treatment completion. Oxygen consumption rate and extracellular acidification revealed an increased metabolic activity of CFs and a switch to glycolytic energy production. These effects were consistent in CFs isolated from the hearts of DOX-treated animals and in naïve CFs exposed to DOX in vitro. The metabolic switch was paralleled with the phenotype change of CFs that upregulated markers of myofibroblast differentiation and the activation of pro-fibrotic signaling. In conclusion, the metabolic switch and activation of CFs anticipate DOX-induced damage and represent a novel target in the early phase of anthracycline cardiomyopathy.

## 1. Introduction

Doxorubicin (DOX) is a highly effective anticancer drug, but its clinical application is hampered by cardiotoxicity that, with time, can evolve into intractable heart failure. Because asymptomatic diastolic dysfunction can be the earliest detectable manifestation, echocardiographic surveillance during anthracycline chemotherapy is recommended [1]. However, there is no clear consensus as to the optimal strategy for preventing and managing chemotherapy-induced cardiotoxicity [2]. The key component that underlies diastolic dysfunction is the excessive deposition of extracellular matrix that contributes to impaired myocardial relaxation and elevated passive stiffness [3]. In the failing heart, the development of fibrosis and possible countermeasures are of substantial clinical interest, but to date, trials of antifibrotic strategies have shown minimal effects at best [4]. Unlike those of the segmental replacement fibrosis and scar formation after myocardial infarction, the timing and molecular triggers of DOX-induced interstitial reactive fibrosis are far less clear. In the advanced stages of anthracycline cardiomyopathy, extensive fibrosis is well recognized, but the understanding of pro-fibrotic mechanisms that operate shortly after drug exposure is limited. This is because cardiac fibroblasts (CFs), one of the largest myocardial cell populations and the source of extracellular matrix, have not been extensively studied in this context. Indeed, most knowledge on the pathogenesis of anthracycline cardiomyopathy is based on the effects of DOX on cardiomyocytes. To fill this gap is also relevant because our perception of the role of CFs in myocardial health and disease evolved from simple mechanical supporters to active players [5,6,7].

The activation of CFs and phenotype switch to myofibroblasts can be linked to the metabolic state of the cell [8]. While a change in energy metabolism caused by DOX is a recognized phenomenon at the cardiomyocyte level [9], the effects of DOX on CF metabolism and the hypothetical link between DOX-induced metabolic changes and CF activity remain to be determined. On this premise, we asked whether the activation of CFs anticipates the onset of DOX-induced early diastolic dysfunction and evaluated the effects of DOX on the activation and metabolism of CFs.

## 2. Methods

### 2.1. In Vivo Treatment with DOX and Animal Protocol

Experimental procedures were carried out in accordance with the national ethical guidelines (Italian Ministry of Health; D.L.vo 26, 4 March 2014) and the guidelines from Directive 2010/63/EU of the European Parliament. Three-month-old female Fischer 344 rats (Charles River Laboratories, Wilmington, MA, USA) were divided into two groups: the DOX group (n = 5) received 6 i.p. injections of 2.5 mg/kg DOX (Adriblastina, Pfizer, Latina, Italy) over a period of 2 weeks; age-matched control rats (n = 5) were saline-injected (CTRL). Female rats were selected in this study since DOX is commonly used in the treatment of women’s breast cancer. Before and after the 2-week treatment with DOX, rats were monitored via transthoracic echocardiography (Vevo 770, VisualSonics, Toronto, ON, Canada) for the assessment of diastolic and systolic dysfunction.

### 2.2. Heart Function

Echocardiography was performed in anesthetized rats (100 mg/kg ketamine and 0.25 mg/kg medetomidine, i.p.). Left ventricle filling pressure was assessed using transmitral pulsed-wave Doppler in a four-chamber view. Mitral valve early wave peak (E wave), atrial wave peak (A wave), E/A ratio, isovolumetric relaxation time (IVRT), and E wave deceleration time (E wave Dec t) were measured. Systolic indices, EF, and fractional shortening (FS) were measured as well [10]. Euthanasia was conducted by an overdose of ketamine (300 mg/kg) and medetomidine (0.75 mg/kg) (3× anesthetic dose) via i.p. administration, followed by heart excision.

### 2.3. Tissue Preparation

Hearts were dissected and placed onto a tissue mold, then covered with OCT cryo-embedding medium and placed in liquid nitrogen. After ensuring the tissue was completely frozen, the tissue block was stored at −80 °C. Tissue sections (10 μm thick) were cut with a cryostat (CM3050 S; Leica Microsystems, Wetzlar, Germany). For molecular biology analysis, samples were directly frozen in liquid nitrogen and stored at −80 °C.

### 2.4. Isolation of Cardiac Primary Fibroblasts

Rat primary cardiac fibroblasts were isolated from healthy (N-CFs) and treated (DOX-CFs) hearts after the first injection of DOX. The hearts were dissected and washed in PBS; fresh tissue was minced and digested for 20 min with 6 mg/mL collagenase type I (Worthington Biochemical Corporation, Worthington, MA, USA) and 5 mg/mL dispase (Gibco, Waltham, MA, USA), 1:1 ratio, in DMEM high glucose supplemented with penicillin (100 U/mL) and streptomycin (100 mg/mL) (Euroclone, Pero, Italy). The digestion was stopped by adding 5 mL of medium supplemented with 10% (*v*/*v*) FBS, and digested hearts were filtered through 70 μm and 40 μm cell strainers. Samples were centrifuged at 1200 rpm for 10 min; the pellet was suspended and placed in DMEM supplemented with 10% (*v*/*v*) FBS, 5% (*v*/*v*) horse serum, 2 mM glutathione, 2.5 U/mL erythropoietin (Sigma-Aldrich, St. Louis, MO, USA), and 3 ng/mL bFGF (Peprotech, Waltham, MA, USA). Twenty-four hours later, floating cells were removed, and adherent cells, mainly composed of fibroblasts, were cultured in the same medium at 37 °C under 5% CO_2_. The fibroblasts used were at passage P1-P3. For fibroblast-to-myofibroblast conversion, 10 ng/mL transforming growth factor-β (TGF-β) stimulation (Sigma-Aldrich) was performed overnight.

### 2.5. Protein Extraction and Western Blot Analysis

For the western blot analysis, 5 × 104 N-CFs and DOX-CFs were seeded in 6-well plates in DMEM-10% FBS and 5% horse serum, grown for 24 h, treated with DOX (0.1 µM) overnight, lysed directly in 2× Laemmli buffer (Bio-Rad Laboratories, Hercules, CA, USA) the next day, resolved by SDS-PAGE under non-reducing or reducing conditions, and blotted onto PVDF membrane transfer packs according to the manufacturer’s instructions (Bio-Rad Laboratories). Tissue samples from CTRL and DOX rats were lysed in buffer (10 mM Tris-HCl, pH 8.0, 150 mM NaCl, 2 mM EDTA, and 1% Nonidet P40) supplemented with a cocktail of protease and phosphatase inhibitors (ThermoFisher Scientific, Waltham, MA, USA) and homogenized on ice using a tissue homogenizer (TissueRuptor; Qiagen, Hilden, Germany). The proteins were separated on SDS-PAGE (30 μg/lane) and transferred onto PVDF membranes [11]. Membranes were incubated with blocking buffer (5% non-fat dry milk in Tween-Tris-buffer saline; Sigma-Aldrich) at room temperature for 1 h and then probed overnight at 4 °C with the following primary antibodies diluted in blocking buffer: anti-connective tissue growth factor (CTGF), anti-galectin-3 (ThermoFisher Scientific), anti-phospho-SMAD 2 (Ser 465/467)/SMAD 3 (Ser 423/425) and SMAD 2/3 (Cell Signaling Technology, Danvers, MA, USA), anti-TGF-β, anti-NADPH oxidase (NOX)-2, anti-matrix metalloproteinase (MMP)-2 and MMP-9, anti-collagen 1, anti-tubulin (Abcam), anti-α-smooth muscle actin (α-SMA), and anti-glyceraldehyde-3-phosphate-dehydrogenase (GAPDH, Sigma-Aldrich). As secondary antibodies, diluted in blocking buffer at room temperature for 1 h, goat anti-rabbit IgG-HRP or goat anti-mouse IgG-HRP (Bethyl Laboratories, Montgomery, TX, USA) were used. Peroxidase activity was measured with an enhanced chemiluminescence kit (Merck Millipore, Burlington, MA, USA). Images were obtained by ChemiDoc-it 500 Imaging System (Bio-Rad Laboratories), and the bands were quantified with the ImageJ 1.52a software.

### 2.6. Immunofluorescence

For immunolabeling, primary fibroblasts were seeded and grown on chamber slides (ThermoFisher Scientific) for 24 h, fixed in 4% paraformaldehyde (Sigma-Aldrich), blocked in 10% normal donkey serum (Jackson ImmunoResearch, West Grove, PA, USA), then incubated overnight (at 4 °C) with anti-α-SMA, anti-vimentin (Novus Biologicals, Abingdon, UK), anti-TGF-β, and collagen 1 (Abcam, Hong Kong, China). Heart sections were incubated at 4 °C overnight with anti-α-SMA, anti-vimentin (Novus Biologicals), and anti-α-sarcomeric actin (Sigma-Aldrich). The primary antibody was revealed using respective anti-mouse IgG/IgM, anti-rabbit IgG, or anti-chicken IgG secondary antibody (Jackson ImmunoResearch). The nuclei were visualized via DAPI staining [12]. Samples were analyzed using a confocal microscope (LSM700, Zeiss, Jena, Germany).

### 2.7. In Vitro Scratch Wound-Healing Assay

Fibroblasts were grown in 6-well plates at a density of approximately 5 × 10^4^ cells/well. After reaching 70–80% confluence, a linear wound was created in the monolayer as per standard methods [13]. Images were captured as a baseline immediately after creating the open wound area and at 1, 6, 18, and 24 h. Images of the cells were obtained using a digital camera (Leica MC120 HD, connected to the inverted microscope Leica DMi1; Leica Mycrosystems, Wetzlar, Germany) and analyzed using image analysis software (ImageJ 1.52e). The extent of wound closure was calculated based on wound area, considered equal to 1 for the area between the monolayer margins at the time of wound formation (0 h).

### 2.8. Mitochondrial Cellular Energetics

Cellular energetics was assessed in live cells using a Seahorse XF24 Analyzer (Agilent Technologies, Santa Clara, CA, USA). An Agilent Seahorse Cell Mito Stress Test Kit was used for assessing mitochondrial stress according to the manufacturer’s instructions (Agilent Technologies). Briefly, 1 × 10^5^ cells/well of N-CFs with or without DOX (0.1 µM) at 6 h, and DOX-CFs, were plated on a Seahorse XF24 cell culture microplate, and mitochondrial function was analyzed using the Seahorse XF24 Analyzer as previously reported [14]. Before the analysis, all the CFs from the experimental group went through the same culture conditions. Multiple parameters were obtained: basal respiration, ATP-linked respiration, maximal and reserve capacities, and non-mitochondrial respiration. Data analysis was performed according to the manufacturer’s instructions. To correct the data interpretation, data normalization was performed via accurate cell counting and measuring of total protein concentration.

### 2.9. Real-Time PCR

Total cellular RNA was isolated using 300 uL of TRIzol LS Reagent (Invitrogen, Waltham, MA, USA) according to the manufacturer’s instructions and treated with a DNase I kit (RQ1 RNase-Free DNase; Promega, Madison, WI, USA). Quantitative real-time PCR (qRT-PCR) was performed in duplicate using the SuperScript III Platinum SYBR Green One-Step qRT-PCR Kit (11736059; Invitrogen) and the CFX96 Real-Time System (Bio-Rad). The transcript levels of enzyme pyruvate kinase M2 (PKM2) and lactate dehydrogenase A (LDHA) were detected, and the housekeeping gene encoding hypoxanthine phosphoribosyltransferase (HPRT) was used as the internal control for mRNA expression studies. Relative expression was calculated using the comparative cycle threshold (Ct) method (2^−ΔΔCt^).

The following primer sequences were used:
PKM2 Fwd: 5′-ATTACCAGCGACCCCACAGAA-3′  Rev: 5′-ACGGCATCCTTACACAGCACA-3′LDHA Fwd: 5′-GCACTAAGCGGTCCCAAAAG-3′  Rev: 5′-ACAGCACCAACCCCAACAAC-3′HPRT Fwd: 5′-TTGTTGGATATGCCCTTGACT-3′  Rev: 5′-CCGCTGTCTTTTAGGCTTTG-3′

### 2.10. Glucose Analog Uptake Assay

For the glucose analog uptake, cells were exposed to 50 µM fluorescent glucose analog 2-NBDG (ThermoFisher Scientific) for 30 min, fixed, and imaged. The density of fluorescence was measured with ImageJ 1.52a software. The background intensity was subtracted to calculate the signal of the cells.

### 2.11. Data Analysis

Results were presented as fold-of-control mean ± standard error of the mean. Data were analyzed by using GraphPad Prism 8.0.2 Software. Significance between two comparisons was determined via Student’s *t*-test and, for multiple comparisons, via one-way ANOVA and Bonferroni’s post-test. All p values are two-sided, and *p* < 0.05 was considered significant.

## 3. Results

### 3.1. Cardiac Function

**Figure 1:**
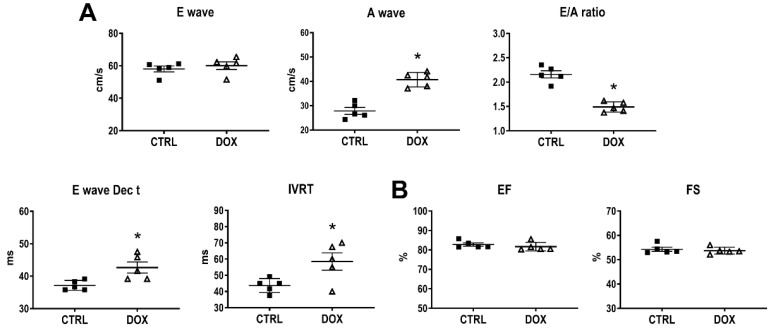
Cardiac function immediately after the completion of DOX treatment. (**A**) Assessment of diastolic function using Doppler measurements of transmitral inflow: peak velocities of the E wave and A wave, the E/A ratio, deceleration time of E wave (E wave Dec t), and isovolumetric relaxation time (IVRT). (**B**) The measurement of systolic function using ejection fractioning (EF) and fractional shortening (FS). * *p* < 0.05 vs. CTRL.

To determine the short-term effects of DOX on cardiac function, echocardiography was conducted immediately after the completion of the treatment cycle (two weeks of repeated exposures to DOX). An interrogation of transmitral inflow via pulsed-wave Doppler imaging showed a significant decrease in the E/A flow velocity ratio, prolongation of E wave Dec t, and an increase in the IVRT, indicating a deficit in diastolic function (Figure 1A). In contrast, systolic function was not changed (Figure 1B). The mild diastolic dysfunction and the unchanged systolic performance corroborate the evidence of a clinical scenario in patients at an early phase of myocardial disease related to chemotherapy [15,16].

### 3.2. Development of the Myocardial Fibrotic Phenotype

**Figure 2:**
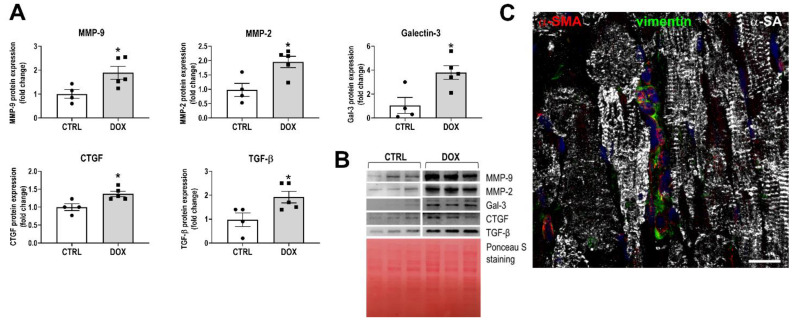
Myocardial fibrosis immediately after the completion of DOX treatment. (**A**) Myocardial expression of matrix metalloproteinase (MMP)-9 and MMP-2, Galectin-3, connective tissue growth factor (CTGF), and transforming growth factor-β (TGF-β) via Western blot. (**B**) Representative Western blot bands and Ponceau staining as loading condition. * *p* < 0.05 vs. CTRL. (**C**) Myocardial section of DOX-treated heart stained with α-smooth muscle actin (α-SMA, red), vimentin (green), and α-sarcomeric actin (α-SA, white); DAPI-labeled nuclei in blue; scale bar 20 μm. The uncropped blots are shown in Figures S1 and S2.

Besides the canonical role of cardiomyocyte damage in the pathogenesis of DOX cardiotoxicity, CFs can also be considered a possible important target [17,18]. To evaluate whether early functional abnormalities were coupled to changes in fibrogenic activity within the myocardium, tissue lysates were obtained at the end of in vivo treatment with DOX. Western blot analysis showed increases in several markers of fibrotic remodeling, such as CTGF, TGF-β, galectin-3, MMP-2, and MMP-9 (Figure 2A,B); the myofibroblast phenotype was observed in the tissue sections, indicating the expression of α-SMA and vimentin (Figure 2C). These data show that the development of the myocardial pro-fibrotic phenotype is a relatively early event in the pathogenesis of anthracycline cardiotoxicity.

### 3.3. Cardiac Fibroblasts from DOX-Injected Animals at the Beginning of Treatment

**Figure 3:**
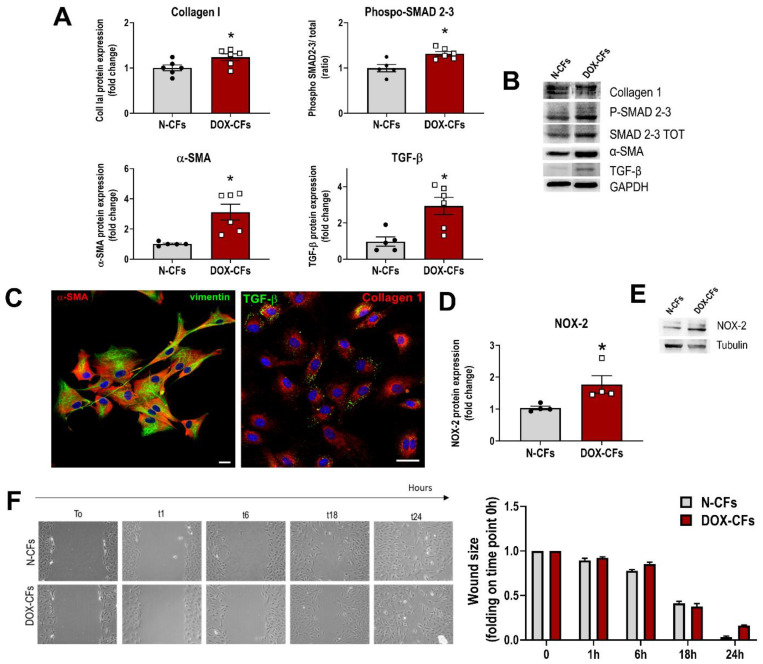
Phenotype of CFs from animals receiving a single DOX injection. (**A**) Protein expression analysis of Collagen I, phospho-SMAD 2-3, α-smooth muscle actin (α-SMA), and transforming growth factor-β (TGF-β) in N-CFs compared with DOX-CFs based on Western blot. (**B**) Representative Western blot bands. (**C**) Primary DOX-CFs stained with α-SMA (red) and vimentin (green) (left panel) or TGF-β (green) and Collagen I (red) (right panel); DAPI-labeled nuclei in blue; scale bars 20 μm (left panel), 40 µm (right panel). (**D**) Protein expression analysis of NADPH oxidase-2 (NOX-2) in N-CFs compared with DOX-CFs based on Western blot. (**E**) Representative Western blot bands. (**F**) Wound-healing assay. For each graph, data points indicate the number of biological replicates. * *p* < 0.05 vs. N-CFs. The uncropped blots are shown in Figures S3–S5.

To test the possibility that the short-term functional and molecular changes were preceded by even earlier effects of DOX on the myocardium, the hearts were examined after only one injection of DOX, well before reaching the established cumulative cardiotoxic dose. Cardiac function was normal, and there was no histological evidence of increased interstitial fibrosis. To study the initial cellular events, CFs were isolated from the hearts of animals that received a single DOX injection (hereafter, these cells are abbreviated as DOX-CFs). DOX-CFs were compared to naïve CFs obtained from normal hearts of age-matched, untreated rats (hereafter, abbreviated as N-CFs). The analysis of cell lysates revealed that DOX-CFs increased the expression of α-SMA and collagen, documenting myofibroblast differentiation and the production of extracellular matrix protein. The enhanced levels of TGF-β and SMAD phosphorylation point out active pro-fibrotic signaling (Figure 3A,B). This cellular phenotype was also evidenced by immunofluorescence in adherent cells (Figure 3C). The enhanced synthesis of TGF-β by DOX-CFs indicates that immediately after the first exposure to DOX, CFs can also impact the surrounding microenvironment and neighboring cells in the autocrine–paracrine manner. Interestingly, DOX-CFs also upregulated NOX-2 (Figure 3D,E), pointing to these cells as an additional intramyocardial source of reactive oxygen in the initial phase of the cardiotoxicity. At the same time, DOX-CFs maintained their functional properties tested in a scratch assay (Figure 3F). Thus, in addition to cardiomyocytes, CFs are immediate myocardial effector cells that swiftly react to systemically administered drugs. These data also suggest that from the very first contact with DOX, CFs can actively contribute to the initial phase of pathological myocardial remodeling.

### 3.4. Naïve Cardiac Fibroblasts Exposed to DOX In Vitro

**Figure 4:**
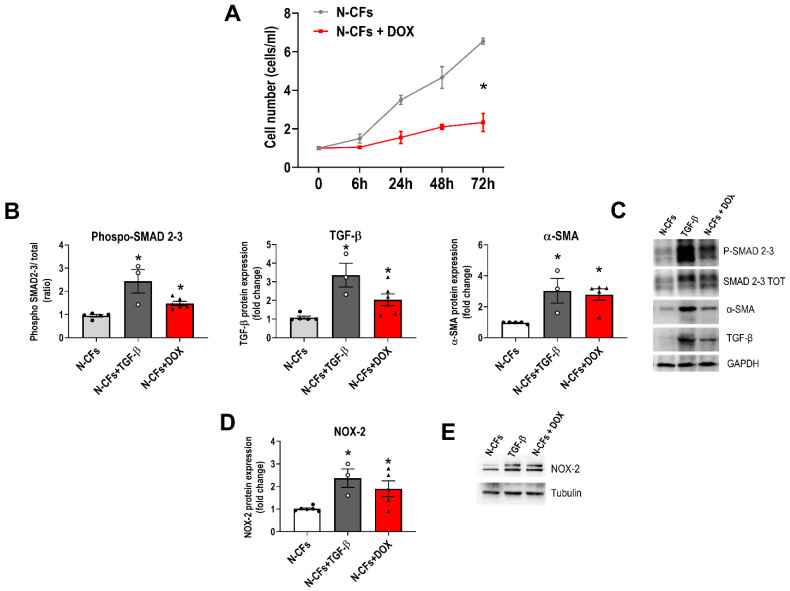
Effect of DOX on primary CFs. (**A**) Growth curves of untreated naïve (N-CFs) and CFs exposed to DOX in vitro. (**B**–**E**) Protein expression analysis of phospho-SMAD 2-3, transforming growth factor-β (TGF-β), α-smooth muscle actin (α-SMA), and NADPH oxidase-2 (NOX-2) in CFs exposed to DOX in vitro (N-CFs+DOX) based on Western blot; representative Western blot bands. TGF-β as a positive control of myofibroblast induction (N-CFs+TGF-β). * *p* < 0.05 vs. N-CFs. For each graph, data points indicate the number of biological replicates. The uncropped blots are shown in Figures S5–S7.

To confirm the possibility that DOX may directly affect the phenotype of CFs, we performed additional experiments where naïve CFs (N-CFs) were exposed to DOX in vitro (N-CFs+DOX). While the cell growth rate was reduced (Figure 4A), exposure to DOX resulted in the augmented acquisition of myofibroblast phenotype (α-SMA expression) and the upregulation of TGF-β (Figure 4B,C). Stimulation of N-CFs with exogenous TGF-β was used as a positive control for cell activation and differentiation. Consistent with the effects of DOX on CFs after the systemic drug administration shown in Figure 3, in vitro exposure to DOX also resulted in the enhanced expression of NOX-2 (Figure 4D,E). Altogether, our in vitro data reinforce the hypothesis that CFs undergo phenotypic reprogramming upon anthracycline exposure.

### 3.5. DOX and Metabolic Profile of Cardiac Fibroblasts

The connection between cell phenotype plasticity and metabolism has been studied in numerous cell types, and there are data suggesting that the remodeling of cellular metabolism can be an initiator of CFs differentiation [8]. In the pathogenesis of anthracycline cardiomyopathy, myocardial energy metabolism and its regulation are considered early targets of DOX. The influence of DOX on energetic networks has been studied in patients, whole perfused hearts, isolated cardiomyocytes, and isolated mitochondria, but the effect on CFs is not known [9]. Therefore, to gain insight into the impact of DOX on the energy metabolism of CFs, two main ATP-producing pathways, mitochondrial respiration and glycolysis, were assessed using the oxygen consumption rate (OCR) and extracellular acidification rate (ECAR) measurements, respectively.

**Figure 5:**
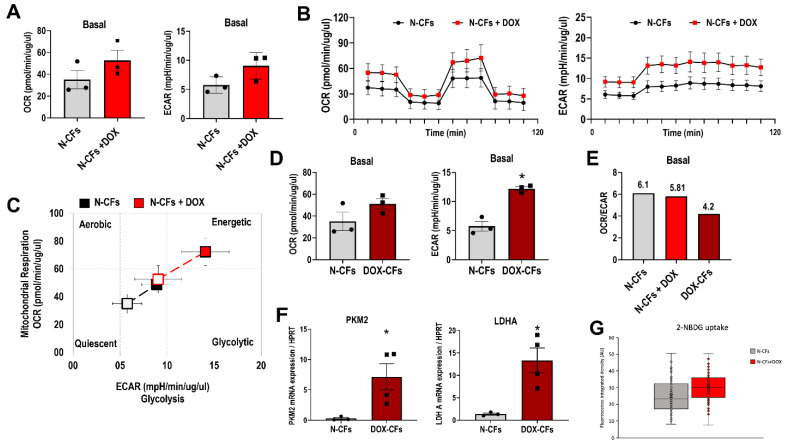
Metabolic changes in CFs. (**A**) Basal oxygen consumption rate (OCR) and basal extracellular acidification rate (ECAR) in untreated and DOX-treated CFs. (**B**) OCR and ECAR profile by Mito stress test in N-CFs and N-CFs+DOX. (**C**) Energy phenotype profile of N-CFs and N-CFs+DOX: baseline phenotype indicated by open markers and stressed phenotype indicated by filled markers. (**D**) Basal OCR and ECAR in N-CFs vs. DOX-CFs. (**E**) Energetic phenotype shown as basal OCR/ECAR ratio. (**F**) Expression of pyruvate kinase (PKM2) and lactate dehydrogenase A (LDHA) in N-CFs and DOX-CFs. * *p* < 0.05 vs. N-CFs. Data points indicate the number of biological replicates (**A**,**D**,**F**). (**G**) Fluorescence intensity of intracellular glucose analog 2-NBDG in N-CFs and N-CFs+DOX.

Following short exposure (6 h) of N-CFs to DOX in vitro (N-CFs+DOX), basal OCR, a measure of mitochondrial oxidative phosphorylation, increased from 35.08 ± 14.50 to 52.61 ± 16.03. In parallel, basal ECAR, which provides a time-resolved view of changes in glycolysis rate, was also enhanced from 5.75 ± 1.42 to 9.05 ± 2.30 (Figure 5A). The changes in OCR and ECAR show the capability of DOX to directly impact the energy metabolism of CFs. Basal, maximal, and spare respiratory capacity, which are metrics of the mitochondrial function derived from the Mito stress test, indicated that efficiency and capacity for mitochondrial ATP synthesis were not compromised (Figure 5B). In N-CFs+DOX, basal and stressed cell energy phenotypes were consistent with augmented energy metabolism with preserved bioenergetic balance between oxidative phosphorylation and glycolysis (Figure 5C). Similar to N-CFs+DOX, cells that were primed in vivo with DOX (DOX-CFs) also showed increased basal oxidative phosphorylation and glycolysis rate (Figure 5D). However, unlike N-CFs+DOX, which maintained a relative contribution of oxidative phosphorylation and glycolysis, DOX-CFs showed an effective decrease in the basal OCR/ECAR ratio (Figure 5E). Moreover, LDHA, which converts pyruvate to lactate necessary to sustain rapid flux through glycolysis, was upregulated. The transcript of the glycolytic enzyme PKM2, which is responsible for the key final step of glycolysis, was also increased (Figure 5F). An increasing trend in the ability to uptake fluorescent glucose analog was also observed (Figure 5G). These data are in line with extracellular flux analysis.

## 4. Discussion

Myocardial homeostasis, which is fundamental for proper cardiac function, is disrupted by noxious stimuli such as chemotherapy, which start the cascade of pathological myocardial remodeling. One aspect of adverse remodeling is reactive fibrosis, which impairs cardiac function via a slow and progressive accumulation of collagen in interstitial and perivascular spaces. In anthracycline cardiotoxicity, reactive fibrosis is the major pathological change. While the research on anthracycline cardiomyopathy has been historically focused on cardiomyocytes, the involvement of other cell types is gaining attention with the possibility that the cardiotoxic effects of DOX can directly involve CFs [18,19]. The results of the present study show that in anthracycline-induced pathological myocardial remodeling, reactive fibrosis is an early and dynamic phenomenon. DOX can trigger reactive fibrosis, and CFs are immediate myocardial effector cells that swiftly react to systemically administered drugs. Because significant myocardial cell loss is not present acutely after DOX exposure, activation of CFs can be considered an independent pathophysiological mechanism rather than a reparative response to the damage.

When activated, fibroblasts undergo differentiation and become phenotypically distinct myofibroblasts with defining features, such as the production of extracellular matrix proteins and the expression of the ⍺-SMA microfilaments. However, this process is more complicated than previously thought, given the heterogeneity of fibroblasts, the complex relationship of fibroblasts and myofibroblasts, and the yet unclear functional differences between various fibroblast states [20,21,22,23]. The unexpected response of CFs to DOX that underwent differentiation instead of growth arrest or cell death emphasizes the role of pathophysiological context in CF fate trajectory. For example, in clearly fibrotic myocardium of *db*/*db* mice, there was no evidence of myofibroblast conversion, underlining that the mechanisms of fibroblast activation in metabolic dysfunction and in myocardial repair are different [24].

Differentiation into myofibroblasts and enhanced extracellular matrix production were not the only features relevant to cardiotoxicity. First, the induction of the pro-fibrotic cytokine TGF-β detected in CFs exposed to DOX could drive autocrine–paracrine signaling and self-propelling positive feedback. Indeed, the phospho-SMAD/SMAD *ratio* was strikingly up, indicating TGF-β pathway activity. In addition to activating quiescent cells, TGF-β could contribute to the persistence of myofibroblasts. This might be the case as we have shown that CFs isolated from the hearts two weeks after DOX treatment had activated phenotype. Second, while the redox cycling of quinone–semiquinone forms of DOX molecule can be a persistent source of reactive species [25], myocardial reactive oxygen species burden can be further increased by CFs. While in non-activated CFs, NOX2 expression was low, consistent with a previous study [26], the exposure to DOX (both in vivo and in vitro) resulted in upregulation of NOX2, the isoform that generates reactive oxygen species and contributes to cardiac fibrosis, hypertrophy, and dysfunction [27].

Our results, obtained with the unique approach to isolate CFs from the hearts after systemic administration of DOX, are also in line with the data from human CFs cell line in vitro, where DOX induced inflammatory and fibrotic responses via the PI3K/Akt and TGF-β/SMAD pathways and increased transcript levels of Toll-like receptor 9, interleukin-1, and cardiac fibrosis markers, such as LGAL3, ACTA2, and TIMP-1 [17,28]. Overall, our data show that following exposure to DOX, CFs can actively contribute to the unfavorable pathophysiological evolution of myocardial disease in its initial phase.

The transition paths from quiescent to activated states have been explored in several pathologies, including cardiomyopathies [29]. One of the prominent features of cell activation is metabolic change. In a non-quiescent state, myofibroblasts increase both aerobic glycolysis and oxidative metabolism [8]. Using extracellular flux analysis, we observed an increase in energy metabolism of CFs exposed to DOX, with amplification of both glycolysis (ECAR) and mitochondrial respiration (OCR). Interestingly, unlike naïve CFs, in which basal OCR/ECAR ratio was not changed after in vitro exposure to DOX, cells that were activated following systemic administration of DOX shifted toward glycolytic pathway, indicating the capacity of DOX to modulate energy metabolism and glycolysis enrichment in CFs. This partitioning of energy production needs further consideration.

Because the amount and the activity of the enzymes and metabolic intermediates can regulate the metabolic flux through a specific pathway, future studies should also aim to determine the candidate metabolic network and specific metabolites involved in the effect of DOX on CF metabolism. The potential effect of DOX on the transport of metabolites across the plasma membrane makes the possible scenario even more complex.

At present, we cannot conclude whether changes in cellular energy transduction through mitochondrial oxidative phosphorylation and substrate-level phosphorylation in glycolysis have driven the phenotypical conversion or vice versa. On the one hand, the acquisition of active phenotype by CFs must be accompanied by metabolic reprogramming to cope with new cellular tasks. On the other hand, myocardial energy metabolism and its regulation are early and most sensitive targets of DOX [9], and there are data indicating metabolic changes as necessary for the activation and differentiation of CFs [8]. In a different context, the effects of DOX on metabolism were reported in lung fibroblasts, when growth arrest and senescence were associated with changes in fatty acid synthesis [30]. Thus, the energy transduction modifications in response to DOX may underlie the process of cellular differentiation.

Several questions arise from our results. One of the remaining problems, which will require a series of dedicated studies, is to determine the molecular mechanism by which DOX promotes CF transformation. Another critical issue to be addressed is a cause–effect relationship between metabolic changes in CFs and the acquisition of myofibroblast phenotype. DOX has the potential to induce a profound transcriptional rewiring of cell metabolism [31] and evoke several ultrastructural changes in the cellular metabolic machinery [32]. In the present study, metabolic effects were present a few hours after exposure to DOX, and myofibroblast phenotype was detected after 48 h. The possibility of the sequential scenario is consistent with the evidence that changes in metabolism not only complement the myofibroblast phenotype but also are required for fibroblast activation and differentiation.

If this is the case, the pharmacologic intervention on the cell metabolism can be envisioned to intercept the activation of the pro-fibrotic cascade at the early step of myofibroblast differentiation.

Several limitations need to be acknowledged. Our study was performed in young and healthy rats. However, given that anthracyclines are generally used in pediatric or mid-age oncologic patients, future studies designed throughout different ages will be of additional value because the fibrotic process builds up naturally with chronologic aging. In view of the long-lasting nature of DOX-related adverse myocardial remodeling, studying the timeline of fibrosis dynamics in more detail can provide useful insights.

In addition, the data on mitochondrion and energy metabolism need to be prudently interpreted in the context of experimental design and mitochondrial heterogeneity between cell types and even within the same cell. The immediate and direct results do not necessarily need to be equivalent to the persistent and cumulative effect of DOX on cardiomyocyte mitochondria [33], and a direct comparison of the mitochondria from CFs and cardiomyocytes may be an option. Also, the extracellular flux analysis should be interpreted with caution because ECAR is not necessarily directly proportional to substrate-level ADP phosphorylation and ECAR to OCR ratios can be influenced by several factors being more indicative than representative. Although the results of the metabolic analysis are promising and hypothesis-generating, we are still far from a clear overview of CF metabolism in response to the anthracycline.

## 5. Conclusions

The activation of CFs anticipates DOX-induced early diastolic dysfunction, before reaching the cumulative cardiotoxic dose. The effect of DOX on the phenotype conversion and metabolic profile in CFs can represent a novel and potentially targetable component of the very early phase of the pathogenesis of anthracycline cardiomyopathy.

## Data Availability

Data is contained within the article and Supplementary Material.

## Supplementary Materials

The following supporting information can be downloaded at: https://www.mdpi.com/article/10.3390/cancers16010053/s1, Figures S1–S7: original images of western blots.
