# Supplementary material for: Initial Phase of Anthracycline Cardiotoxicity Involves Cardiac Fibroblasts Activation and Metabolic Switch
Source: Cancers (Basel). 2023 Dec 21;16(1):53. doi: 10.3390/cancers16010053 (PMC10778158; doi:10.3390/cancers16010053)

Ponceau S staining

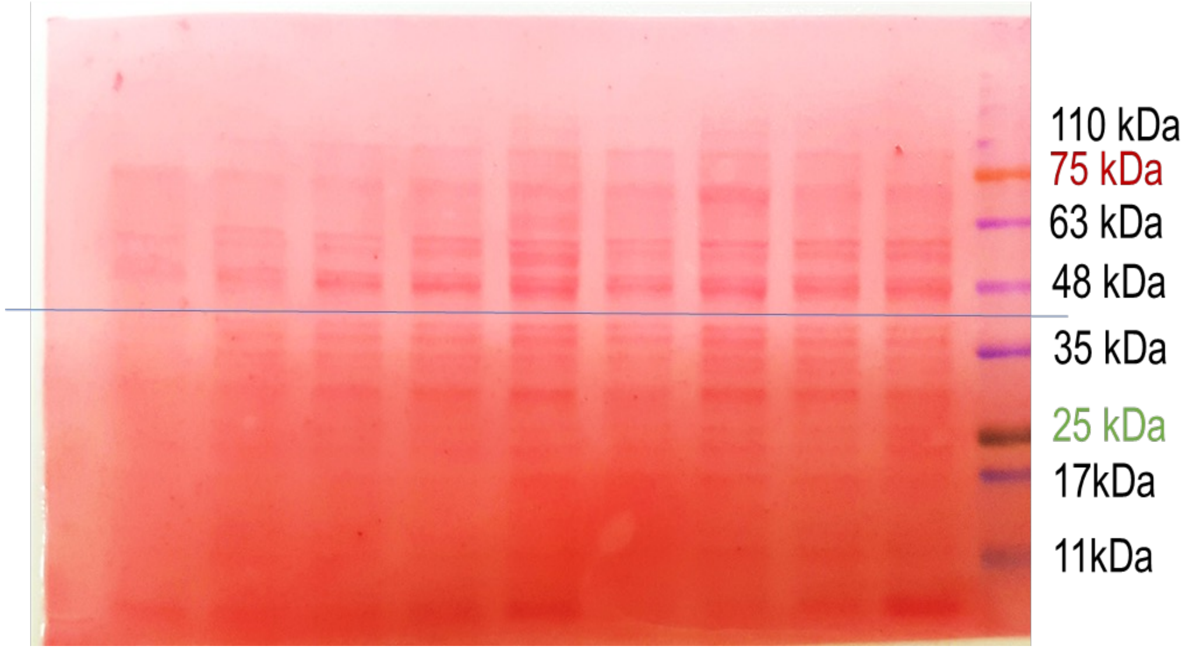

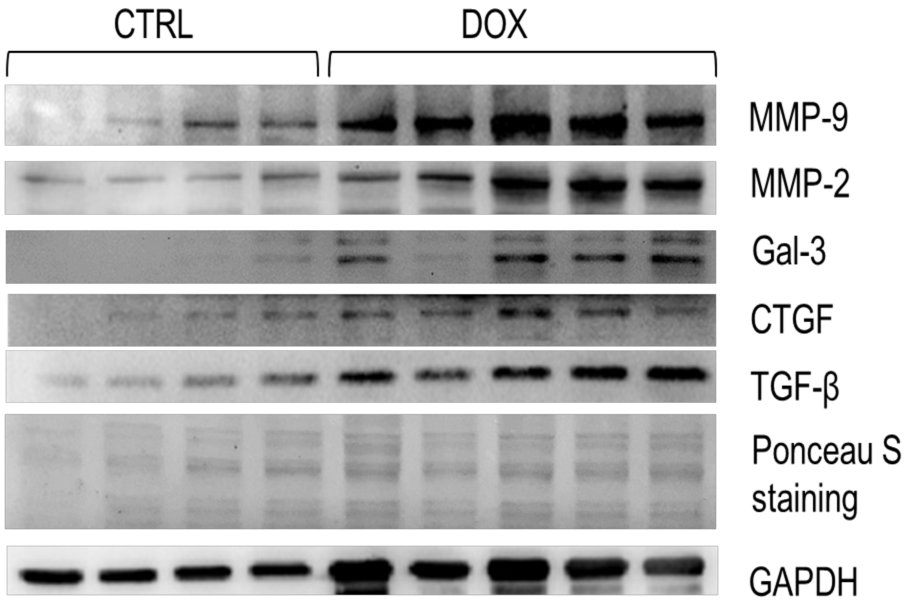

Figure S1: original blots of Figure 2

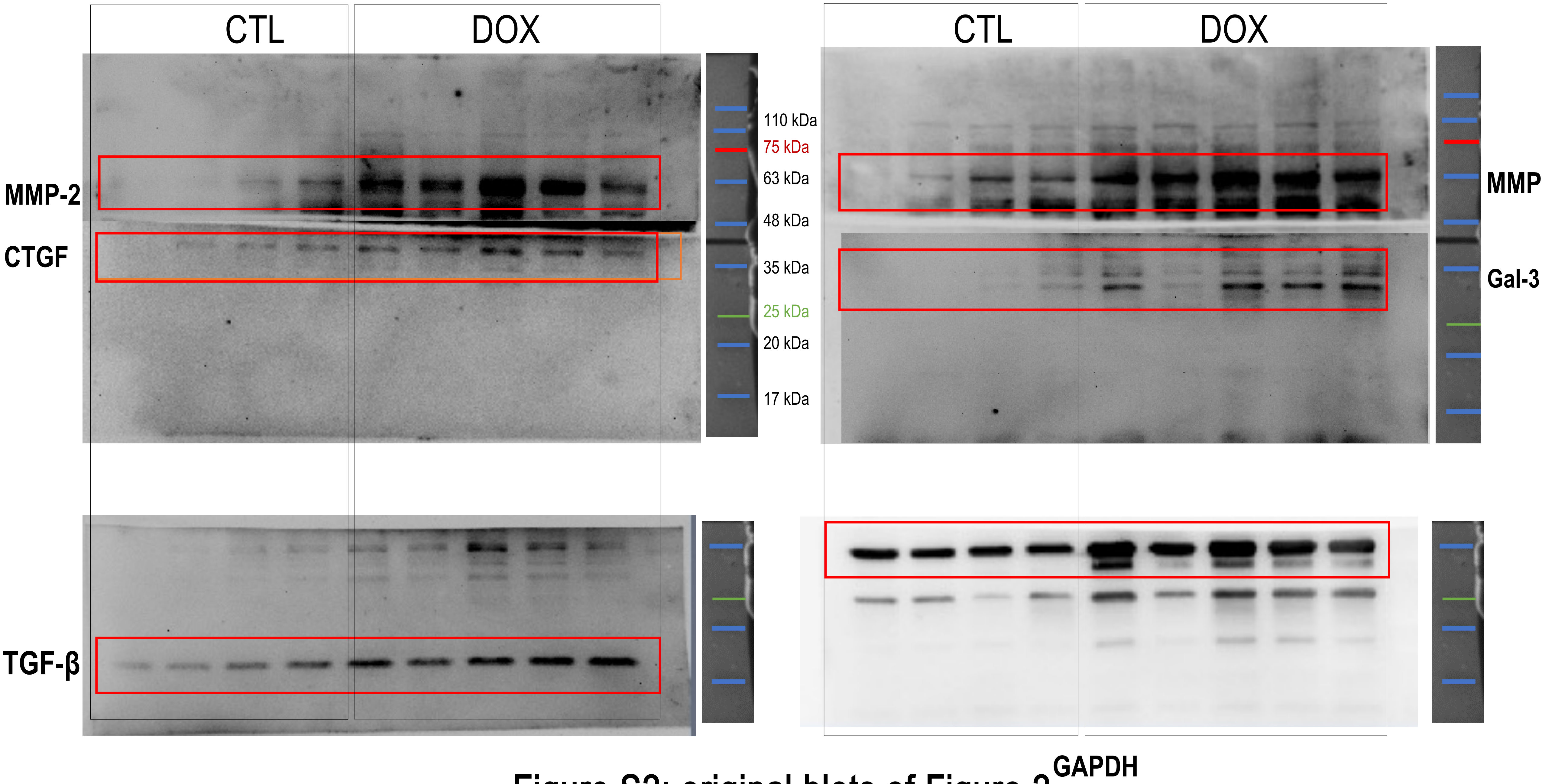

Figure S2: original blots of Figure 2

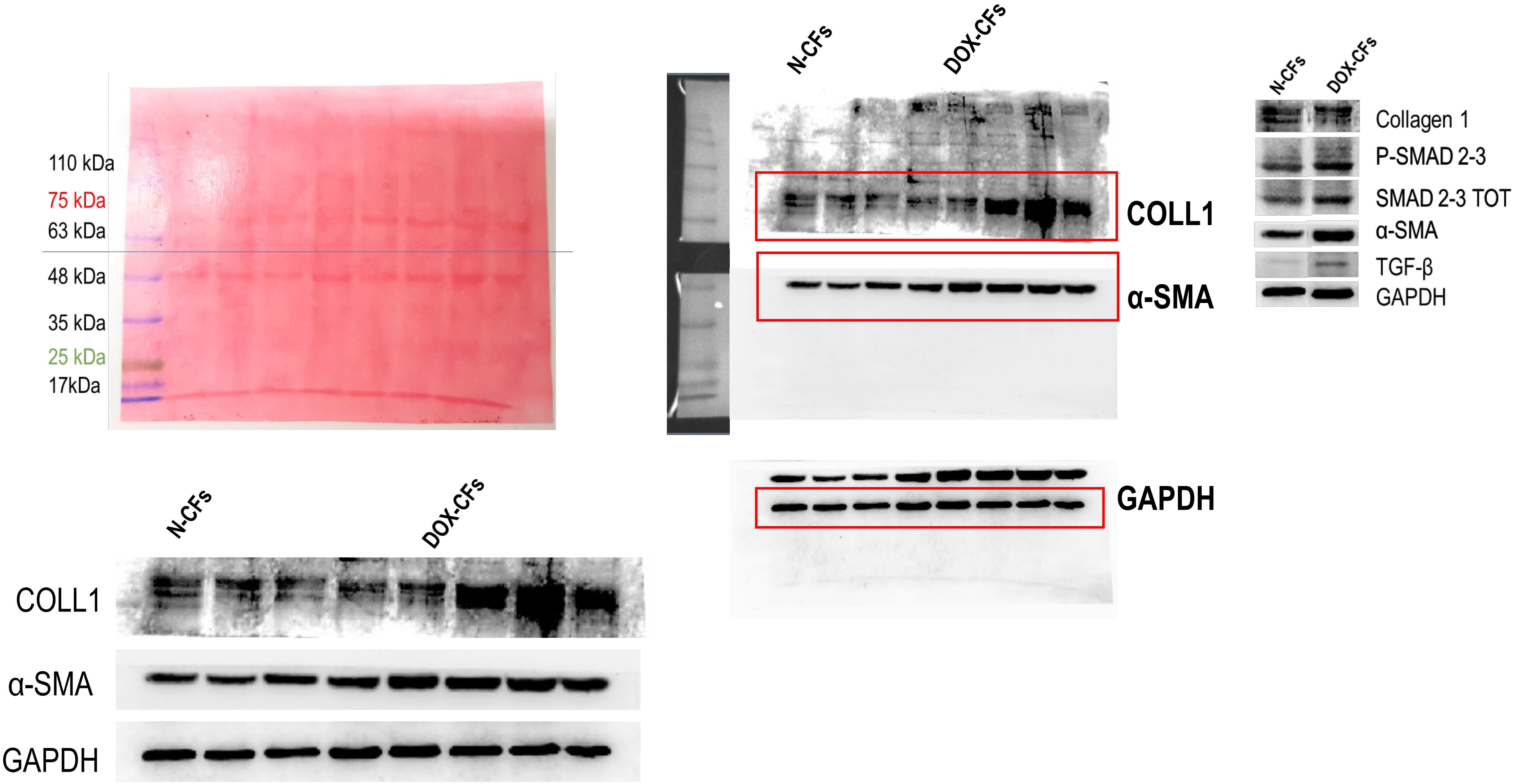

Figure S3: original blots of Figure 3

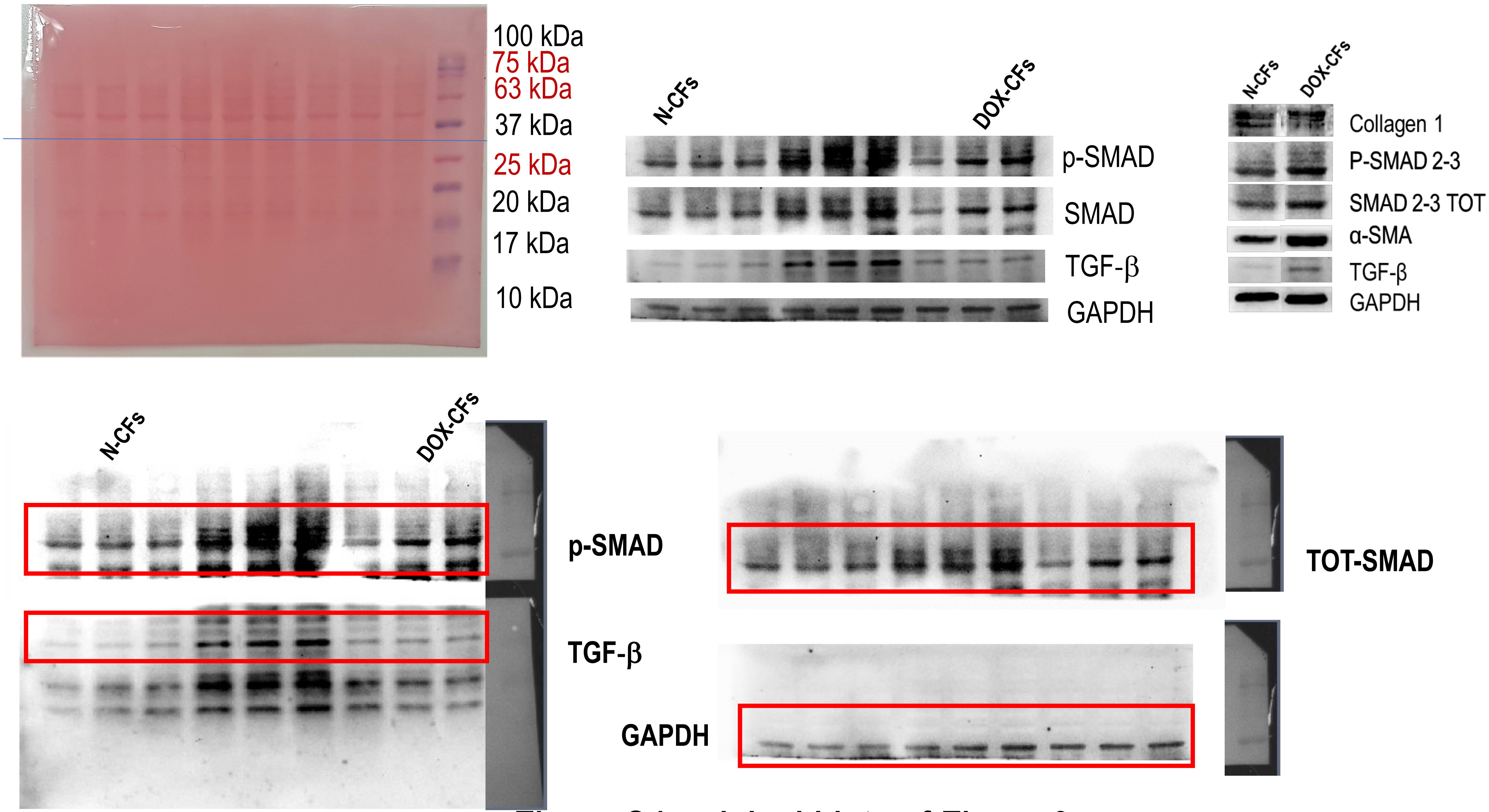

Figure S4: original blots of Figure 3

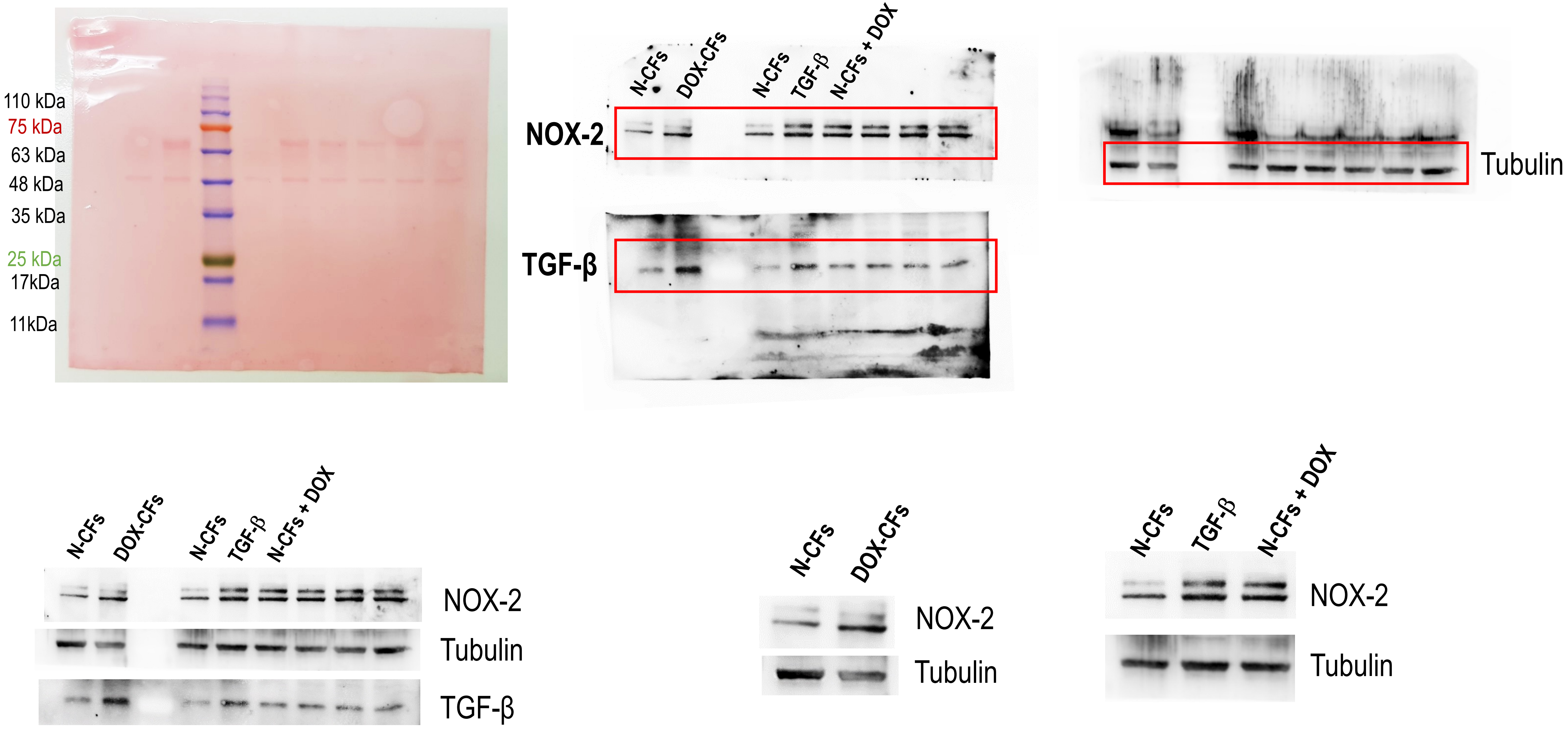

Figure S5: original blots of Figure 3 and Figure 4

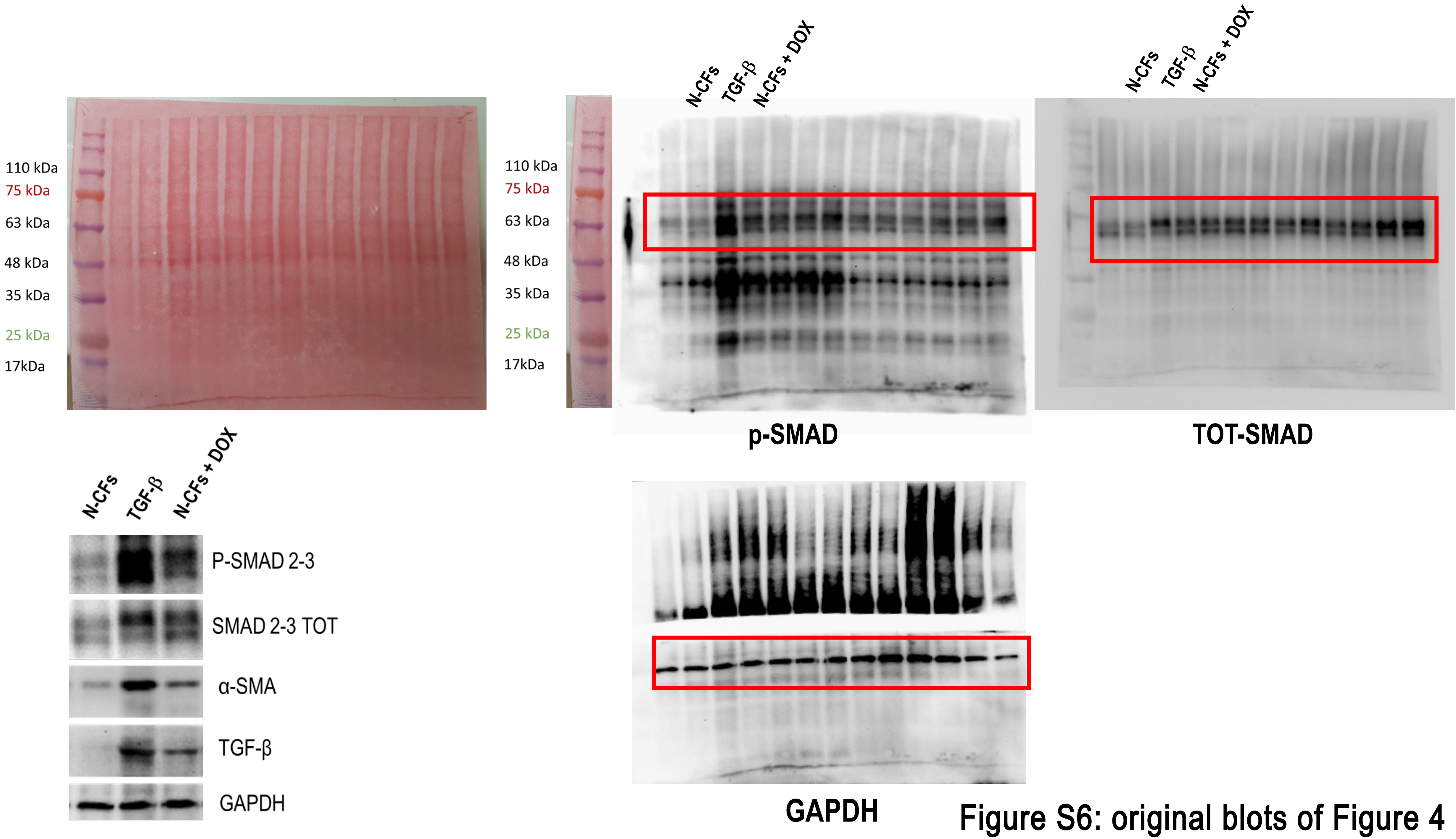

Figure S6: original blots of Figure 4

Figure S7: original blots of Figure 4

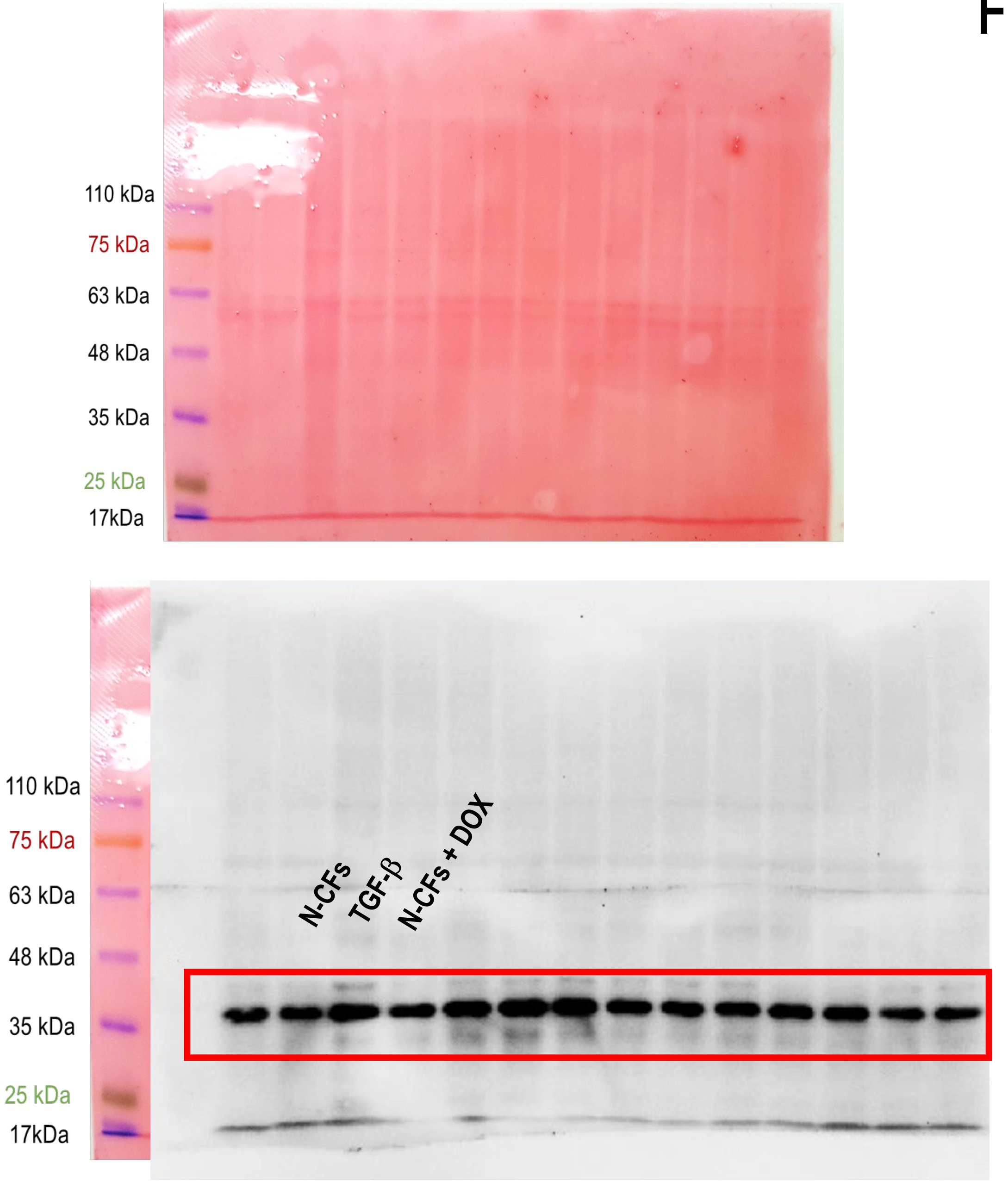

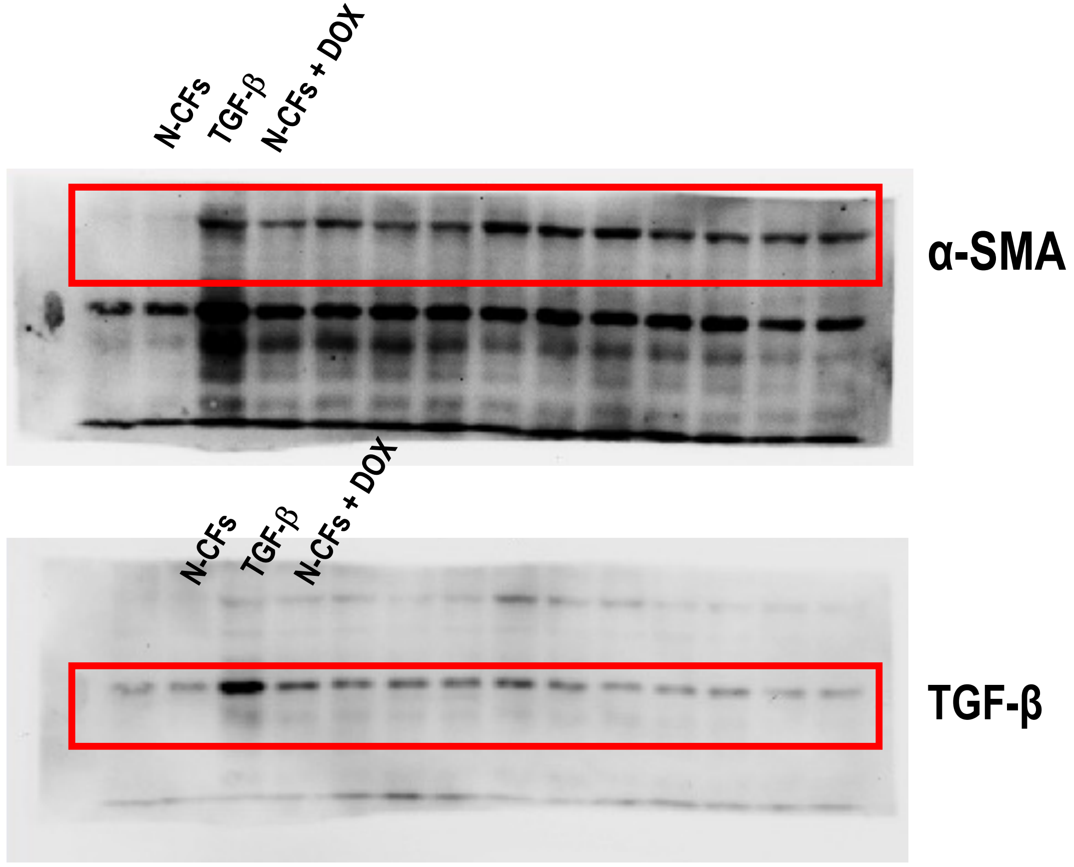

Supplement: Supplementary file 1 [file cancers-16-00053-s001.zip › cancers-2663455-supplementary.pdf]
